# High‐intensity focused ultrasound ablation combined with transcatheter arterial chemoembolization improves long‐term efficacy and prognosis of primary liver cancer

**DOI:** 10.1002/jcla.23633

**Published:** 2020-10-25

**Authors:** Meng Sun, Pengyan Shang, Jiangtao Bai, Shanfeng Li, Meng Li

**Affiliations:** ^1^ Interventional Vascular Surgery Affiliated Hospital of Hebei University Baoding Hebei China; ^2^ Baoding maternal and Child Health Hospital Baoding Hebei China

**Keywords:** efficacy, primary liver cancer, prognosis, TACE combined with HIFU regimen

## Abstract

**Background:**

To investigate the clinical efficacy of high‐intensity focused ultrasound (HIFU) combined with transcatheter arterial chemoembolization (TACE) in the treatment of primary liver cancer (PLC) and its effect on the prognosis of patients.

**Methods:**

A total of 132 patients with PLC admitted to our hospital were selected for the study, among whom 68 patients received TACE combined with HIUF and were assigned to the observation group (OG), whereas the remaining 54 patients were treated with TACE alone and were assigned to the control group (CG). The factors influencing the patients’ prognosis were also evaluated by multivariate analysis.

**Results:**

The total effective rate of the OG was 83.82%, which was significantly higher than that of 55.56% of the CG (*P* < .05). No significant difference was found in incidence of adverse reactions between the two groups (*P* > .05). After treatment, the increases of CD3+, CD4+, CD4+/CD8+, and NK cells in the OG were more significant than those in the CG (*P* < .05). However, the decrease of CD8+ cells was more significant in the OG than that in the CG (*P* < .05). The 3‐year survival rate of patients in the OG was 61.76%, which was significantly higher than that of 40.74% in the CG (*P* < .05).

**Conclusion:**

The application of TACE combined with HIFU is effective in treating PLC, which can prolong the life expectancy and improve the prognosis of patients with PLC without increasing the incidence of adverse reactions.

## Graphical abstract

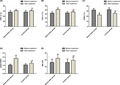

## 1 INTRODUCTION

Primary liver cancer (PLC) is one of the most common malignant gastrointestinal tumors in clinical practice. The incidence of liver cancer in China accounts for >5% of the global incidence. In addition, PLC‐related mortality in China is up to 45%, ranking the first worldwide.^1^ For patients with PLC, surgical resection is the only intervention clinically recognized as having therapeutic capacity. However, PLC has a high degree of malignancy and an insidious onset, such that approximately 70%–80% of patients are in the advanced stage before the manifestation of symptoms. This generally leads to a loss of the optimal timing for surgical intervention.^2^, ^3^

Transcatheter arterial chemoembolization (TACE) is the mainstream clinical treatment for advanced PLC and the most recognized non‐surgical treatment currently.^4^, ^5^, ^6^ Although its antitumor effect has been acknowledged, TACE cannot kill tumor cells completely. This is complicated by the fact that repeated treatment can induce local ischemia and hypoxia at the embolized site, leading to an increased synthesis of vascular endothelial growth factor and hypoxia‐inducing factor, thus aggravating liver damage.^7^, ^8^ The further complication is that several adverse reactions occur during treatment, resulting in unsatisfactory clinical effects.^9^, ^10^ In recent years, radiofrequency ablation has been widely applied in the clinical treatment of liver cancer due to its small damage, exact curative effect, and high safety.^11^ As a new interventional therapy, high‐intensity focused ultrasound (HIFU) ablation has attracted much attention in terms of its therapeutic value in malignant tumors.^12^, ^13^ For example, Vidal‐Jove^14^ considered HIFU to be a potentially effective and safe method for the treatment of malignant tumors, with a survival advantage in the treatment of unresectable pancreatic cancer. Zhang^15^ reported that HIFU could achieve complete tumor necrosis even if the lesion was located near the major liver vessels and could be safely used to ablate tumors adjacent to the major blood vessels. Although HIFU has been proven to be more valuable in the treatment of pancreatic and liver cancers, few studies have been conducted on HIFU ablation combined with TACE in the treatment of PLC.

Therefore, in this study, we analyzed the clinical efficacy of TACE combined with HIFU in monitoring the liver function of patients with PLC, and examined the long‐term survival rate of patients treated with this regimen and the main factors that affect prognosis, so as to provide a reference for the development of individualized treatment plan and prognosis for patients with PLC.

## 2 MATERIALS AND METHODS

### 2.1 General information

A total of 132 patients with PLC admitted to our hospital between June 2014 and August 2016 were selected as the study subjects. Patients who were treated with TACE combined with HIUF were assigned in the observation group (OG) (n = 68) and those who were treated with TACE alone were assigned to the control group (CG) (n = 54). There were 44 males and 24 females in the OG, aged 37‐63 years (average age 51.71 ± 11.26 years). In the CG, there were 38 males and 16 females, aged 41‐65 years (average age 50.29 ± 10.27 years).

### 2.2 Inclusion and exclusion criteria

Inclusion criteria: All the patients met the diagnostic criteria of PLC through puncture biopsy or according to the criteria of European Association for the Study of the Liver.^16^ They neither received any prior treatment before admission nor surgical indication or antisurgical treatment. In addition, the patients and their family members were fully informed, and informed consent form was signed and provided by them. This study has been approved by the Ethics Committee of Affiliated Hospital of Hebei University. The study methodologies conformed to the standards set by the Declaration of Helsinki.

Exclusion criteria: Patients with the Child–Pugh grade of the liver function that was greater than grade C (including grade C); patients with refractory ascites; patients with complications from other malignant tumors; patients with other cardiovascular, cerebrovascular, liver, or renal diseases; patients taking other drugs during treatment without the consent of the attending physician which affected the therapeutic effects; patients with hepatic artery‐portal vein fistula; patients with a complete blockage of the main portal vein; patients with serious diseases of heart, brain, liver, lungs, kidneys, or other organs; patients with other malignant tumors or serious metabolic diseases or mental diseases; and patients withdrew from the study.

### 2.3 Treatment

After admission, patients in the CG received TACE alone. The specific methods were as follows: The Seldinger technique was used to percutaneously puncture the femoral artery into the hepatic artery, and the donor artery of the tumor was determined by DSA perfusion angiography, followed by the conduction of TACE. The number of times TACE was needed was determined every 3‐4 weeks depending on the blood supply artery, with 1‐3 times per person. Patients in the OG underwent ultrasound ablation on the 7th day after TACE treatment as those in the CG. JC200HIFU treatment system was adopted, with the parameters set as follows: frequency: 0.96 MHz, focal length: 134 mm, focus: (length × width) 6 × 2 mm^2^, and the focal field sound intensity range: 4000‐12 000 W/cm^2^. Under general anesthesia, the treatment area was cleaned, prepared for skin treatment, and the location and size of the tumor were determined according to the imaging examination results. Next, the treatment position was adjusted and artificial pleural effusion was performed according to the patient's condition. After establishing the safety sound channel, the patient was subjected to HIFU ablation treatment. Intraoperatively, the gray scale changes in the target tissues, skin, and adjacent organs were closely monitored. The treatment was conducted from point to line, line to surface, and deep to shallow, with the coverage exceeding 1 cm of the tumor boundary.

### 2.4 Efficacy evaluation

The study subjects were examined by abdominal CT or MRI before and 1 month after treatment. According to the volume change of tumor focus, the tumor volume reduction rate was measured and calculated as follows: volume reduction rate = (pretreatment volume‐post‐treatment volume)/pretreatment volume × 100%. The ablation effect was evaluated according to the modified Response Evaluation Criteria in Solid Tumors (mRECIST) criteria^17^: (a) complete response (CR): tumor lesion shrinkage and necrosis of >50%; (b) partial response (PR): tumor lesion shrinkage and necrosis of 25%–50%; (c) stable disease (SD): tumor lesion shrinkage and necrosis of <25%; and (d) progressive disease (PD): tumor lesions increased by >50%, total effective rate = CR + PR + SD. After 3 months of treatment, the adverse reactions of the two groups were evaluated.

### 2.5 Patient follow‐up

All the 132 patients were followed up by telephone or interview every 3 months for 3 years until August 2019. Three months after treatment is the exact testing time of the immune function after treatment. The total survival period was considered from the first day after surgery to the last follow‐up or death.

### 2.6 Statistical analysis

The data were statistically analyzed by Statistic Package for Social Science (SPSS) 23.0 (IBM Corp, Armonk, New York, USA). The measurement data were expressed as mean ± standard deviation (mean ± SD), and the counting data were represented by [n (%)]. A *t* test of independent samples was employed for intergroup comparison of measurement data, and a paired t test was applied for intragroup comparison. The counting data within the group were expressed as the number of cases (%), and the chi‐square test was used for comparison. *P* < .05 indicated a statistically significant difference.

## 3 RESULTS

### 3.1 General information

There was no statistically significant difference in the general information between the two groups, including gender, age, smoking history, drinking history, body mass index (BMI), residence, education level, working status, tumor location, tumor diameter, alpha‐fetoprotein (AFP) level, and KPS score between the two groups (*P* > .05, Table 1).

**Table 1:** General information of patients in the two groups [n(%)]/(x ± SD)

Categories	The OG (n = 68)	The CG (n = 54)	t/χ^2^	*P*
Gender			0.438	.508
Male	44 (64.71)	38 (70.37)		
Female	24 (35.29)	16 (29.63)		
Age (y)	51.71 ± 11.26	50.29 ± 10.27	0.719	.474
Smoking history			0.492	.622
Yes	36 (52.94)	31 (57.41)		
No	32 (47.06)	23 (42.59)		
Drinking history			0.278	.598
Yes	41 (60.29)	30 (55.56)		
No	27 (39.71)	24 (44.44)		
BMI (kg/m^2^)	23.56 ± 2.78	22.87 ± 3.31	1.251	.213
Residence			0.163	.687
Rural	39 (57.35)	29 (53.7)		
Urban	29 (42.65)	25 (46.3)		
Education level			0.327	.568
High school and below	51 (75)	38 (70.37)		
High school or above	17 (25)	16 (29.63)		
Working status			0.128	.72
No	17 (25)	12 (22.22)		
Yes	51 (75)	42 (77.78)		
Tumor location			0.624	.43
Right half liver	38 (55.88)	34 (62.96)		
Left half liver	30 (44.12)	20 (37.04)		
Tumor diameter	16.45 ± 5.64	15.97 ± 6.86	0.424	.672
AFP level			0.525	.469
≤400ng/ml	27 (39.71)	18 (33.33)		
>400ng/ml	41 (60.29)	36 (66.67)		
KPS score	64.72 ± 7.15	65.86 ± 6.89	0.79	.43

### 3.2 Comparison of clinical efficacy between the OG and the CG

After treatment, there were 6 cases (8.82%) of CR, 36 (52.94%) of PR, 15 (22.06%) of SD, and 11 (16.18%) of PD in the OG, with the total effective rate of 83.82%. In the CG, 3 cases (5.56%) presented with CR, 16 (29.63%) with PR, 11 (20.37%) with SD, and 24 (44.44%) with PD, and the total effective rate was 55.56%. Therefore, the total effective rate of the OG was significantly higher than that of the CG (*P* < .05) (Table 2).

**Table 2:** Comparison of clinical efficacy between the CG and the OG [n(%)]

Categories	The OG (n = 68)	The CG (n = 54)	χ^2^value	*P*
CR	6 (8.82)	3 (5.56)	12.7	.005
PR	36 (52.94)	16 (29.63)		
SD	15 (22.06)	11 (20.37)		
PD	11 (16.18)	24 (44.44)		
Total effective rate	57 (83.82)	30 (55.56)	11.76	.001

### 3.3 Adverse reactions in the two groups

In the OG, there were 3 cases (4.41%) of skin burns, 2 (2.94%) of liver function injury, and 6 (8.82%) of nausea and loss of appetite, and the incidence of total adverse reactions was 16.18%. In the CG, there was 1 case (1.85%) of skin burns, 3 (5.56%) of liver function injury, and 5 (9.26%) of nausea and loss of appetite, and the total incidence of adverse reactions was 16.67%. The above data indicated that there was no significant difference in the incidence of skin burns, liver function injury, nausea, and loss of appetite between the OG and CG (*P* > .05) (Table 3).

**Table 3:** Comparison of the incidence of toxic and side effects between the OG and the CG [n(%)]

Categories		The OG (n = 68)	The CG (n = 54)	χ^2^ value	*P*
Skin burn		3 (4.41)	1 (1.85)	0.622	.430
Liverfunction injury	2 (2.94)	3 (5.56)	0.523	.469	
Nausea and loss of appetite		6 (8.82)	5 (9.26)	0.523	.469
Total incidence rate		11 (16.18)	9 (16.67)	0.301	.583

### 3.4 Changes of serum CD3+, CD4+, CD8+, CD4+/CD8+, and NK cells before and after treatment in the two groups

**Figure 1:**
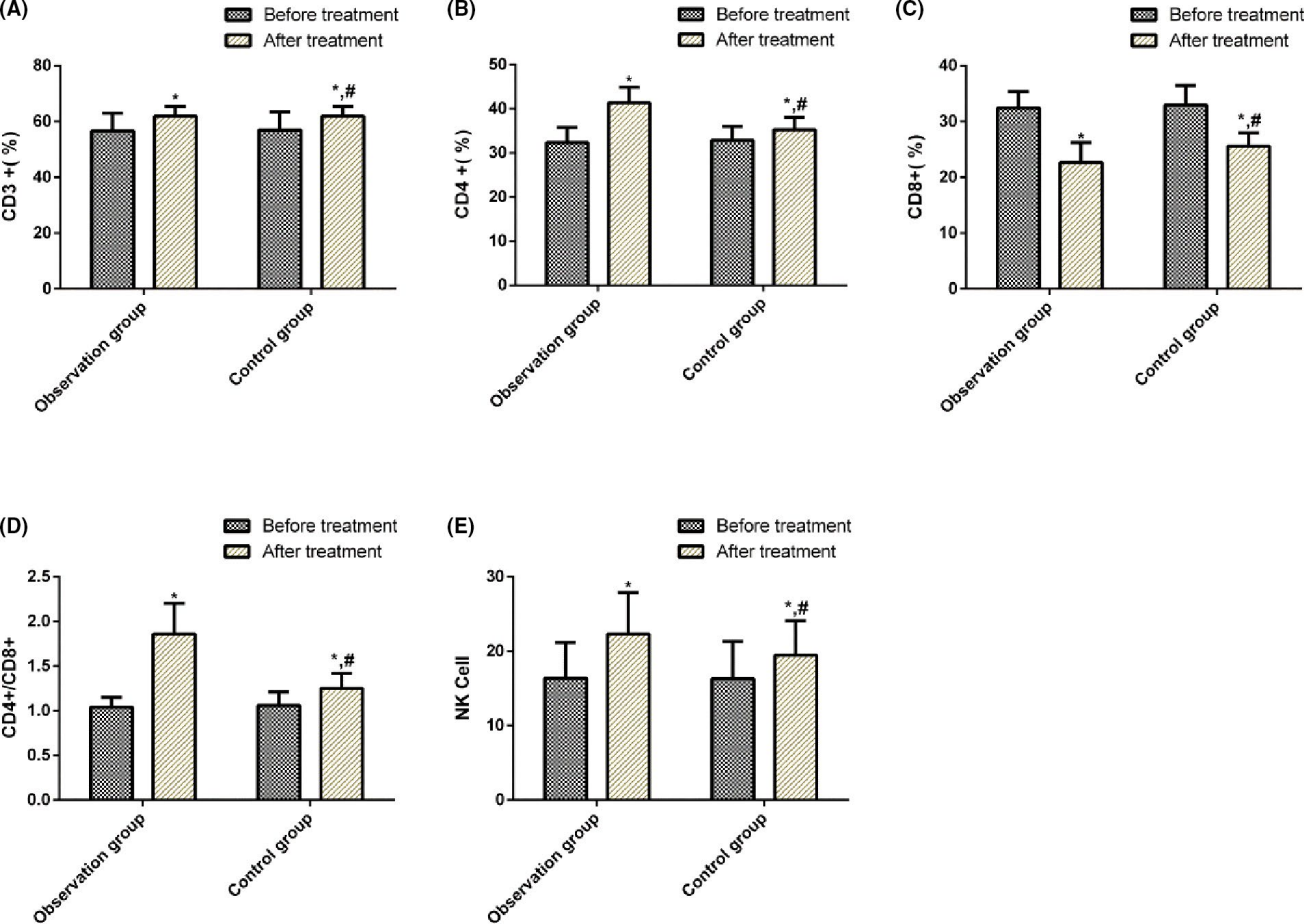
Comparison of serum CD3+, CD4+, CD8+, CD4+/CD8+, and NK cells in the CG and the OG before and after treatment. Note: *indicates *P* < .05 compared with before the treatment period and #indicates *P* < .05 compared with the CG after treatment

There were no significant differences in serum CD3+, CD4+, CD8+, and CD4+/CD8+ between the two groups before treatment (*P* > .05). However, after treatment, the serum CD3+, CD4+, CD4+/CD8+, and NK cells were all significantly increased in the two groups (*P* < .05), and the concentration of CD8+ was significantly lower than that before treatment (*P* < .05). The OG presented significantly higher concentrations of serum CD3+, CD4+, CD4+/CD8+, and NK cells (*P* < .05) and a lower concentration of CD8+ than the CG (Table 4, Figure 1).

**Table 4:** Comparison of serum CD3+, CD4+, CD8+, CD4+/CD8+, and NK cells (x ± SD)

	Groups	CD3+(%)		CD4+ (%)		CD8+ (%)		CD4+/CD8+		NK cells (%)
	Before treatment	After treatment	Before treatment	After treatment	Before treatment	After treatment	Before treatment	After treatment	Before treatment	After treatment
The OG (n = 68)	56.59 ± 6.37	77.85 ± 6.38*	32.35 ± 3.42	41.37 ± 3.52*	32.43 ± 2.96	22.65 ± 3.58*	1.04 ± 0.11	1.86 ± 0.34*	16.37 ± 4.78	22.31 ± 5.57*
The CG (n = 54)	56.97 ± 6.46	61.87 ± 3.51*	32.88 ± 3.12	35.27 ± 2.83*	32.91 ± 3.54	25.59 ± 2.37*	1.06 ± 0.15	1.25 ± 0.17*	16.29 ± 5.03	19.45 ± 4.63*
t value	0.325	17.580	0.884	10.350	0.816	5.196	0.849	12.040	0.090	3.031
P	0.746	<0.001	0.379	<0.001	0.416	<0.001	0.397	<0.001	0.929	0.003

Note:

* indicates *P* < .05 compared with before treatment.

### 3.5 Three‐year survival of patients in the OG and CG after surgery

**Figure 2:**
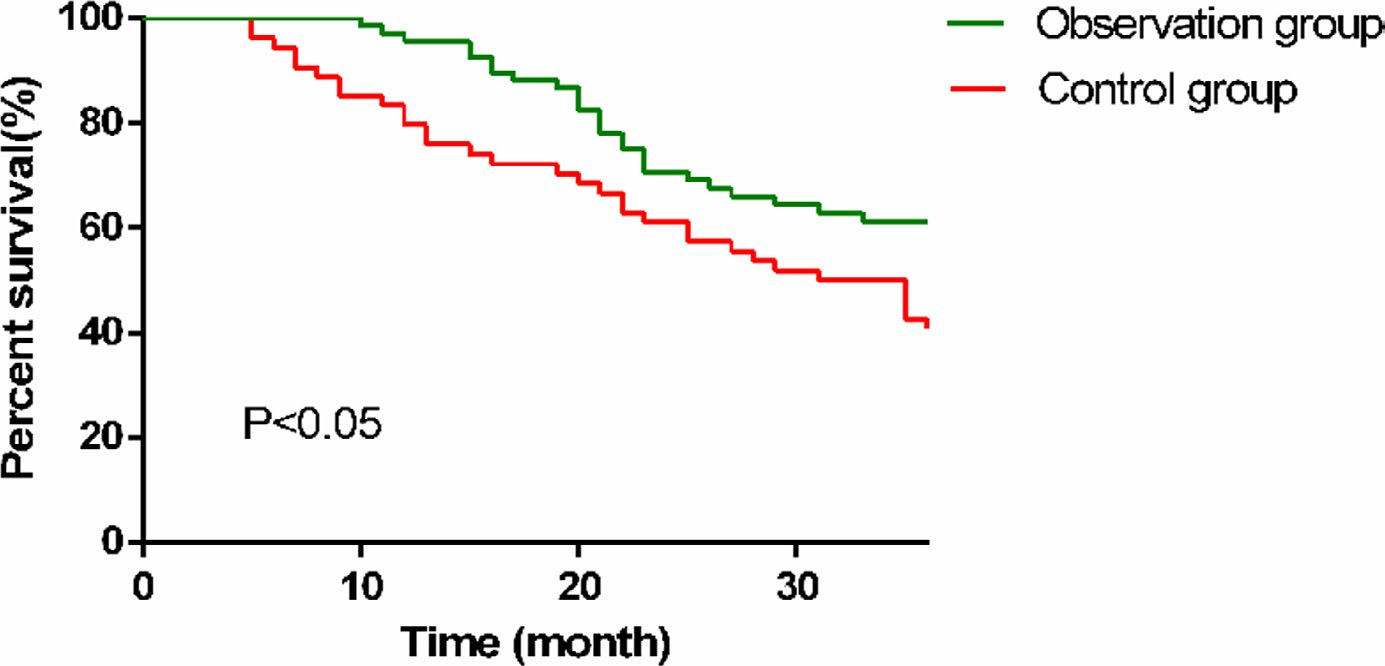
Three‐year survival of patients in the two groups. The 3‐year survival rate in the OG (61.76%) was significantly higher than that in the CG (40.74%) (*P* < .05)

We performed statistical analysis on the patient's 3‐year survival after surgery. All the patients were successfully followed up, and no patient was lost to follow‐up. In the 3 years, 58 patients died and 64 survived, with the survival rate of 52.46%. Among them, 26 patients died and 42 survived in the OG, with the survival rate of 61.76%. However, 32 patients died and 22 patients survived in the CG, with the survival rate of 40.74%. K–M survival curve was plotted according to the 3‐year survival of the patients in the two groups, and it was found that the 3‐year survival rate of the OG was higher than that of the CG, with a statistically significant difference (*P* < .05) (Figure 2).

### 3.6 Univariate analysis on the prognosis of patients with PLC

The clinical data of patients in the survival and death groups were collected for univariate analysis. It was observed that there was no difference in terms of gender, smoking history, tumor location, WBC, PLT, or tumor location between the two groups (*P* > .05). However, a significant difference was observed in the lesion size, bilirubin level, treatment methods, combined with collateral portal vein circulation, and AFP (*P* < .05) (Table 5).

**Table 5:** Univariate analysis of prognosis of MM patients (x ± SD)

Factors	The survival group (n = 64)	The death group (n = 58)	χ^2^ value	*P*
Age (y)			0.728	.467
<60	51 (79.69)	43 (74.14)		
≥60	13 (20.31)	15 (25.86)		
Gender			0.587	.444
Male	45 (70.31)	37 (63.79)		
Female	19 (29.69)	21 (36.21)		
Smoking history			0.612	.434
Yes	33 (51.56)	34 (58.62)		
No	31 (48.44)	24 (41.38)		
AFP level			9.946	.002
≤400 ng/mL	32 (50)	13 (22.41)		
>40 0ng/mL	32 (50)	45 (77.59)		
Tumor location			0.081	.776
Right half liver	37 (57.81)	35 (60.34)		
Left half liver	27 (42.19)	23 (39.66)		
WBC			0.229	.632
<4 × 10^9^/L	27 (42.19)	22 (37.93)		
≥4 × 10^9^/L	37 (57.81)	36 (62.07)		
PLT			0.698	.404
<100 × 10^9^/L	39 (60.94)	31 (53.45)		
≥100 × 10^9^/L	25 (39.06)	27 (46.55)		
Lesion size			5.334	.021
≤3 cm	42 (65.63)	26 (44.83)		
>3 cm	22 (34.38)	32 (55.17)		
Bilirubin			0.618	.432
≤1.5 mg/dL	21 (32.81)	23 (39.66)		
>1.5 mg/dL	43 (67.19)	35 (60.34)		
collateral portal vein circulation			6.229	.013
Yes	33 (51.56)	17 (29.31)		
No	31 (48.44)	41 (70.69)		
Treatment methods			9.239	.002
TACE	20 (31.25)	34 (58.62)		
HIFU combined with TACE	44 (68.75)	24 (41.38)		

### 3.7 Multivariate analysis of prognosis and survival of patients with PLC

Indicators with differences in univariate analysis were included in the assignment (the assignment tables are shown in Table 6), followed by multivariate Cox regression analysis. The results indicated that lesion size, bilirubin level, treatment methods, combined with portal vein collateral circulation, and AFP were independent risk factors affecting the prognosis of HCC (*P* < .05) (Table 7).

**Table 6:** Assignment table

Factors	Assignments
Lesion size	≤3 = 0; >3 = 1
Bilirubin	≤1.5mg/dL = 0; >1.5mg/dL = 1
Treatment methods	HIFU combined with TACE = 0; TACE = 1
Combined with portal vein collateral circulation	Yes = 0; No = 1
AFP	≤400 = 0; >400 = 1

**Table 7:** Multivariate analysis of prognosis in patients with PLC

	B	SE	Wald	P	HR	95% CI
Lesion size	0.21	0.137	3.215	0.732	1.31	0.79‐1.81
Bilirubin	0.73	0.264	6.832	0.007	2.23	1.37‐3.12
Treatment methods	3.74	1.868	4.20	0.469	1.13	0.06‐4.77
collateral portal vein circulation	0.65	0.138	5.643	0.021	1.97	1.42‐2.65
AFP	2.69	0.543	22.711	0.872	0.77	0.25‐5.81

## 4 DISCUSSION

PLC is one of the most common malignant tumors. According to clinical studies, people aged 40‐50 years are the most susceptible to the occurrence of PLC, and its incidence in males is significantly higher than that in females.^18^, ^19^ To date, the etiology of PLC has not been determined; however, some scholars believe that PLC is related to liver cirrhosis, viral hepatitis, aflatoxin, and environmental factors.^20^, ^21^ Its clinical symptoms include pain and digestive tract symptoms, and in severe cases, liver cancer metastasis results in systemic symptoms, which seriously affects patients’ quality of life and health.^22^, ^23^ However, with the deterioration associated with liver cancer, far fewer than 20% of patients can receive surgical treatment. Therefore, it is of great significance to explore new treatment methods for patients with PLC.^24^, ^25^

TACE is a kind of chemotherapy that selectively embolizes tumor blood vessels through catheters, thereby making tumor cells ischemic and necrotic through vascular embolization and exerting an antitumor role. TACE has been proven to inhibit vascular invasion, delay tumor progression, and prolong patient survival.^26^ HIFU ablation is a new type of interventional therapy with the advantages of high penetrability and focusability of ultrasound on tissues, which can focus on the tumor target tissues with low intensity with body surface penetrability, and increase the temperature of local target tissues to >65°C within a short time, leading to the destruction of tumor cells due to coagulative necrosis. As a noninvasive antitumor interventional therapy, HIFU ablation has been recognized for its efficacy and safety in the treatment of liver cancers.^27^, ^28^, ^29^ The results of this study showed that the clinical efficacy of the OG was significantly higher than that of the CG, and there was no significant difference in the incidence of skin burns, impairment of liver function, and abdominal discomfort between the two groups (*P* < .05). Siyu^30^ observed that the effective and total effective rates of TACE combined with HIFU were 61.1% and 94.4%, respectively, which were significantly higher than those in the CG. According to Luo,^31^ TACE combined with HIFU for PLC had a higher overall response rate and a lower incidence of damage to normal liver tissues, which could completely kill tumor cells and reduce postoperative local recurrence and metastasis rate with fewer adverse reactions. This was consistent with the results of our study that TACE combined with HIFU ablation was a feasible option with considerable efficacy.

The occurrence, progression, and metastasis of PLC are closely related to the immune function of the body.^32^ Cellular immunity is the main mechanism of antitumor effect, with T‐lymphocyte exerting vital regulatory effects in the tumor immune response. Although the T‐lymphocyte subsets are one of the most important cell groups in the immune system and are the paramount immune cells that maintain the internal environment of the immune system and mediate cellular immunity,^33^ CD4+, as a helper T‐lymphocyte, can secrete cytokines that enhance the ability of CD8+ and NK cells to kill tumors.^34^ CD8 + T lymphocytes can directly kill tumor cells and inhibit cellular and humoral immunity.^35^ The dynamic balance between the two plays an important role in maintaining immune function, only when the CD4+/CD8+ ratio is normal can normal antitumor effects be produced.^36^ NK cells are mainly distributed in the blood circulatory system and lymphatic organs. Under the action of chemotactic mediators, they can quickly enter a lesion site through the blood and kill tumor cells. Hence, a decrease in the NK cell activity usually indicates a distant metastasis of the tumor.^37^ The majority of patients with PLC are often found in the middle and late stages, with reduced T lymphocytes, imbalanced T‐lymphocyte subgroup, and decreased immune function. In this study, the serum CD3+, CD4+, CD4+/CD8+, and NK cells in the two groups were significantly increased after treatment (*P* < .05), and CD8+ concentration was significantly lower than that before treatment (*P* < .05). The intergroup comparison demonstrated that after treatment, serum concentrations of CD3+, CD4+, NK cells, and CD4+/CD8+ in the OG were significantly higher than those in the CG (*P* < .05), whereas the CD8+ concentration was significantly lower than that in the CG, indicating that TACE combined with HIFU improved the immune function of patients with PLC to a greater extent and enhanced the antitumor ability of the body. Maybe through the regulation improve the T lymphocytes, balance the T‐lymphocyte subgroup, and increased immune function. Subsequently, we statistically analyzed the 3‐year survival of the patients and found that the 3‐year survival rate of the OG was significantly higher than that of the CG, suggesting that TACE combined with HIFU improves the survival of patients with PLC. Finally, multivariate Cox regression analysis was performed, which demonstrated that lesion size, bilirubin level, treatment methods, combined with portal vein collateral circulation, and AFP were independent factors for PLC patients. This suggests that they can be used as clinical indicators to evaluate the prognosis of PLC.

However, this study has certain limitations. First, due to the limited experimental conditions, narrowed sample size, and the insufficient time span for survival analysis, more cases need to be collected for long‐term follow‐up study. Secondly, the correlation between other relevant clinical indicators and PLC has not been discussed in depth. This should be explored in subsequent trials.

In conclusion, TACE combined with HIFU regimen has a better efficacy in treatment of PLC. It can also prolong the survival of patients without increasing the incidence of adverse reactions, which is clinically useful.

## CONFLICT OF INTERESTS

None.

## References

[1] Wang X , Yu Q , Yu P , et al. Surgical treatment of huge hepatocarcinoma with invasion or severe adhesion of diaphragm using the technique of orthotopic liver resection. Hepatogastroenterology. 2015;62(137):153‐156. 25911887

[2] Zhou J , Sun H‐C , Wang Z , et al. Guidelines for diagnosis and treatment of primary liver cancer in China (2017 Edition). Liver Cancer. 2018;7(3):235‐260. 30319983 10.1159/000488035 PMC6167671

[3] Orcutt ST , Anaya DA . Liver resection and surgical strategies for management of primary liver cancer. Cancer Control. 2018;25(1):1073274817744621. 10.1177/1073274817744621 PMC5933574 29327594

[4] Dev A , Sood A , Choudhury SR , Karmakar S . Paclitaxel nanocrystalline assemblies as a potential transcatheter arterial chemoembolization (TACE) candidate for unresectable hepatocellular carcinoma. Mater Sci Eng C Mater Biol Appl. 2020;107:110315. 31761231 10.1016/j.msec.2019.110315

[5] Arizumi T , Ueshima K , Minami T , et al. Effectiveness of sorafenib in patients with transcatheter arterial chemoembolization (TACE) refractory and intermediate‐stage hepatocellular carcinoma. Liver Cancer. 2015;4(4):253‐262. 26734579 10.1159/000367743 PMC4698649

[6] Farinati F , Vanin V , Giacomin A , et al. BCLC stage B hepatocellular carcinoma and transcatheter arterial chemoembolization: a 20‐year survey by the Italian Liver Cancer group. Liver Int. 2015;35(1):223‐231. 25074434 10.1111/liv.12649

[7] Huo YR , Eslick GD . Transcatheter arterial chemoembolization plus radiotherapy compared with chemoembolization alone for hepatocellular carcinoma: a systematic review and meta‐analysis. JAMA Oncol. 2015;1(6):756‐765. 26182200 10.1001/jamaoncol.2015.2189

[8] Hirooka M , Hiraoka A , Ochi H , et al. Transcatheter arterial chemoembolization with or without radiofrequency ablation: outcomes in patients with Barcelona Clinic Liver Cancer stage B hepatocellular carcinoma. AJR Am J Roentgenol. 2018;210(4):891‐898. 29412017 10.2214/AJR.17.18177

[9] Zhao X , Zhang X , Kuang R , Jiang Y , Jiang L , Fu M . Effect of bifid‐triple viable capsule combined with reduced glutathione injection on liver function and intestinal flora in patients with primary hepatocellular carcinoma after transcatheter hepatic arterial chemoembolization. Int J Clin Exp Med. 2018;11(10):10865‐10872.

[10] He L , Xu Q , Chen L , Chen R . A meta‐analysis of arsenic trioxide combined with transcatheter arterial chemoembolization for treatment of primary hepatic carcinoma. Evid Based Complement Alternat Med. 2016;2016. 10.1155/2016/3428370 PMC4921631 27382405

[11] Mirza AN , Fornage BD , Sneige N , et al. Radiofrequency ablation of solid tumors. Cancer J. 2001;7(2):95‐102. 11324771

[12] Feijoo ERC , Sivaraman A , Barret E , et al. Focal high‐intensity focused ultrasound targeted hemiablation for unilateral prostate cancer: a prospective evaluation of oncologic and functional outcomes. Eur Urol. 2016;69(2):214‐220. 26164416 10.1016/j.eururo.2015.06.018

[13] Hsiao Y‐H , Kuo S‐J , Tsai H‐D , Chou M‐C , Yeh G‐P . Clinical application of high‐intensity focused ultrasound in cancer therapy. J Cancer. 2016;7(3):225. 26918034 10.7150/jca.13906 PMC4747875

[14] Vidal‐Jove J , Perich E , del Castillo MA . Ultrasound guided high intensity focused ultrasound for malignant tumors: the Spanish experience of survival advantage in stage III and IV pancreatic cancer. Ultrason Sonochem. 2015;27:703‐706. 26044461 10.1016/j.ultsonch.2015.05.026

[15] Zhang L , Zhu H , Jin C , et al. High‐intensity focused ultrasound (HIFU): effective and safe therapy for hepatocellular carcinoma adjacent to major hepatic veins. Eur Radiol. 2009;19(2):437. 18795303 10.1007/s00330-008-1137-0

[16] Sacco R , Gadaleta‐Caldarola G , Galati G , Lombardi G , Mazza G , Cabibbo G . European Association for the Study of the Liver Hepatocellular Carcinoma summit 2014: old questions, new (or few) answers? Future Oncol. 2014;10(10):1719‐1721. 25303051 10.2217/fon.14.102

[17] Therasse P , Arbuck SG , Eisenhauer EA , et al. New guidelines to evaluate the response to treatment in solid tumors. European Organization for Research and Treatment of Cancer, National Cancer Institute of the United States, National Cancer Institute of Canada. J Natl Cancer Inst. 2000;92(3):205‐216. 10655437 10.1093/jnci/92.3.205

[18] Akinyemiju T , Abera S , Ahmed M , et al. The burden of primary liver cancer and underlying etiologies from 1990 to 2015 at the global, regional, and national level: results from the Global Burden of Disease Study 2015. JAMA Oncol. 2017;3(12):1683‐1691. 28983565 10.1001/jamaoncol.2017.3055 PMC5824275

[19] Valery PC , Laversanne M , Clark PJ , Petrick JL , McGlynn KA , Bray F . Projections of primary liver cancer to 2030 in 30 countries worldwide. Hepatology. 2018;67(2):600‐611. 28859220 10.1002/hep.29498 PMC5832532

[20] Kudo M , Kitano M , Sakurai T , Nishida N . General rules for the clinical and pathological study of primary liver cancer, nationwide follow‐up survey and clinical practice guidelines: the outstanding achievements of the Liver Cancer Study Group of Japan. Dig Dis. 2015;33(6):765‐770. 26488173 10.1159/000439101

[21] Zimmermann E , Berentzen TL , Gamborg M , Sørensen TI , Baker JL . Sex‐specific associations between birth weight and adult primary liver cancer in a large cohort of D anish children. Int J Cancer. 2016;138(6):1410‐1415. 26506514 10.1002/ijc.29900

[22] Fukui N , Golabi P , Otgonsuren M , de Avila L , Bush H , Younossi Z . Hospice care in Medicare patients with primary liver cancer: the impact on resource utilisation and mortality. Aliment Pharmacol Ther. 2018;47(5):680‐688. 29314093 10.1111/apt.14484

[23] Petrick JL , Freedman ND , Demuth J , et al. Obesity, diabetes, serum glucose, and risk of primary liver cancer by birth cohort, race/ethnicity, and sex: Multiphasic health checkup study. Cancer Epidemiol. 2016;42:140‐146. 27148890 10.1016/j.canep.2016.04.009 PMC4899157

[24] Toesca DA , Osmundson EC , von Eyben R , Shaffer JL , Koong AC , Chang DT . Assessment of hepatic function decline after stereotactic body radiation therapy for primary liver cancer. Pract Radiat Oncol. 2017;7(3):173‐182. 28343896 10.1016/j.prro.2016.10.003

[25] Song H , Qiao F , Shao M . Research advances in traditional Chinese medicine treatment for primary liver cancer. J Clin Hepatol. 2016;1:174‐177.

[26] Chao Y , Chung YH , Han G , et al. The combination of transcatheter arterial chemoembolization and sorafenib is well tolerated and effective in A sian patients with hepatocellular carcinoma: Final results of the START trial. Int J Cancer. 2015;136(6):1458‐1467. 25099027 10.1002/ijc.29126

[27] Palermo G , Totaro A , Sacco E , et al. Hifu as first line salvage therapy in prostate cancer local relapse after radical prostatectomy: 4‐year follow‐up outcomes. Minerva Urol Nefrol. 2016;69(1):93‐100. 28009150 10.23736/S0393-2249.16.02696-5

[28] Shaw C , Rivens I , Civale J , et al. OP21. 10: Technical and safety considerations for high intensity focused ultrasound (HIFU) non‐invasive placental vascular occlusion. Ultrasound Obstet Gynecol. 2016;48:121‐122.26482947

[29] Seip R , Sanghvi N , Carlson R , Chaluisan R , Morris A , Carol M , inventors; Google Patents, assignee. System, apparatus and method for high‐intensity focused ultrasound (hifu) and/or ultrasound delivery while protecting critical structures. 2018.

[30] Siyu F , Ren R , Wang M , Chen Y , Jiang B , Zhang Q . Clinical observation of TACE combined with HIFU in the treatment of middle and advanced primary He‐patocellular Carcinoma. China Pharmacy. 2015;26(35):4978‐4980.

[31] Luo Y , Jiang Y . Comparison of efficiency of TACE plus HIFU and TACE alone on patients with primary liver cancer. J Coll Physicians Surg Pak. 2019;29(5):414‐417. 31036108 10.29271/jcpsp.2019.05.414

[32] Cheng J‐W , Shi Y‐H , Fan J , et al. An immune function assay predicts post‐transplant recurrence in patients with hepatocellular carcinoma. J Cancer Res Clin Oncol. 2011;137(10):1445. 21809031 10.1007/s00432-011-1014-0 PMC11827982

[33] Melichar B , Touskova M , Solichova D , Kralickova P , Kopecký O . CD4+ T‐lymphocytopenia and systemic immune activation in patients with primary and secondary liver tumours. Scand J Clin Lab Invest. 2001;61(5):363‐370. 11569483 10.1080/003655101316911404

[34] DuPage M , Bluestone JA . Harnessing the plasticity of CD4+ T cells to treat immune‐mediated disease. Nat Rev Immunol. 2016;16(3):149. 26875830 10.1038/nri.2015.18

[35] Alp ÖS , Radbruch A . The lifestyle of memory CD8+ T cells. Nat Rev Immunol. 2016;16(4):271. 10.1038/nri.2016.32 26996198

[36] Hughes RA , May MT , Tilling K , et al. Long terms trends in CD4: CD8+ ratio+: CD8+ ratio cell counts, CD8: CD8+ ratio+: CD8+ ratio cell counts, and the CD4: CD8+ ratio+: CD8+ ratio: CD8+ ratio. AIDS. 2018;32(10):1361‐1367. 29851663 10.1097/QAD.0000000000001848 PMC5991182

[37] Ruscetti M , Leibold J , Bott MJ , et al. NK cell–mediated cytotoxicity contributes to tumor control by a cytostatic drug combination. Science. 2018;362(6421):1416‐1422. 30573629 10.1126/science.aas9090 PMC6711172

